# Directly mining a fungal thermostable α-amylase from Chinese Nong-flavor liquor starter

**DOI:** 10.1186/s12934-018-0878-y

**Published:** 2018-02-22

**Authors:** Zhuolin Yi, Yang Fang, Kaize He, Dayu Liu, Huibo Luo, Dong Zhao, Hui He, Yanling Jin, Hai Zhao

**Affiliations:** 1 Meat-processing Application Key Laboratory of Sichuan Province, College of Pharmacy and Biological Engineering, Chengdu University, Chengdu, China; 2 Key Laboratory of Environmental and Applied Microbiology, Chinese Academy of Sciences, Chengdu, China; 3 Environmental Microbiology Key Laboratory of Sichuan Province, Chengdu Institute of Biology, Chinese Academy of Sciences, No. 9 Section 4, Renmin Nan Road, Chengdu, 610041 Sichuan People’s Republic of China; 4 Liquor Making Bio-Technology & Application of Key Laboratory of Sichuan Province, Bioengineering College, Sichuan University of Science & Engineering, Zigong, China; 5 Wuliangye Group, Yibin, China; 6 Department of Liquor Making Engineering, Moutai College, Renhuai, China

**Keywords:** Chinese Nong-flavor liquor starter, Thermostable, Fungal α-amylase, Starch degradation, Solid-state simultaneous saccharification and fermentation

## Abstract

**Background:**

Chinese Nong-flavor (NF) liquor is continuously and stably produced by solid-state fermentation technology for 1000 years, resulting in enrichment of special microbial community and enzymes system in its starter. Based on traditional culture-dependent methods, these functional enzymes are hardly obtained. According to our previous metatranscriptomic analysis, which identifies plenty of thermostable carbohydrate-active enzymes in NF liquor starter, the aim of this study is to provide a direct and efficient way to mine these thermostable enzymes.

**Results:**

In present study, an alpha-amylase (NFAmy13A) gene, which showed the highest expression level of enzymes in starch degradation at high temperature stage (62 °C), was directly obtained by functional metatranscriptomics from Chinese Nong-flavor liquor starter and expressed in *Pichia pastoris*. NFAmy13A had a typical signal peptide and shared the highest sequence identity of 64% with α-amylase from *Aspergillus niger*. The recombinant enzyme of NFAmy13A showed an optimal pH at 5.0–5.5 and optimal temperature at 60 °C. NFAmy13A was activated and stabilized by Ca^2+^, and its half-lives at 60 and 70 °C were improved significantly from 1.5 and 0.4 h to 16 and 0.7 h, respectively, in the presence of 10 mM CaCl_2_. Meanwhile, Hg^2+^, Co^2+^ and SDS largely inhibited its activity. NFAmy13A showed the maximum activity on amylopectin, followed by various starches, amylose, glycogen, and pullulan, and its specificity activity on amylopectin was 200.4 U/mg. Moreover, this α-amylase efficiently hydrolyzed starches (from corn, wheat, and potato) at high concentrations up to 15 mg/ml.

**Conclusions:**

This study provides a direct way to mine active enzymes from man-made environment of NF liquor starter, by which a fungal thermostable α-amylase (NFAmy13A) is successfully obtained. The good characteristics of NFAmy13A in degrading starch at high temperature are consistent with its pivotal role in solid-state fermentation of NF liquor brewing. This work would stimulate mining more enzymes from NF liquor starter and studying their potentially synergistic roles in NF liquor brewing, thus paving the way toward the optimization of liquor production and improvement of liquor quality in future.

**Electronic supplementary material:**

The online version of this article (10.1186/s12934-018-0878-y) contains supplementary material, which is available to authorized users.

## Background

Chinese liquor accounts for more than one-third of all spirits consumed in the world according to the International Wine and Spirit Research Group. Chinese liquor is produced by a solid-state simultaneous saccharification and fermentation (SSF) in an environment-friendly way for 1000 years, and Nong-flavor (NF, also called strong aroma type) liquor accounts for more than 70% of Chinese liquor production [1, 2]. Its manufacture mainly contains two steps: 4 months of liquor starter (daqu) production and 40–45 days of alcohol solid-state fermentation [3, 4]. Special and stable microbial communities are enriched during the NF liquor starter production process [5], and in the mature NF liquor starter [6]. These microbes would secret various enzymes, e.g., amylase, acid protease, cellulase, lipase, and esterase, to efficiently hydrolyze carbohydrates and proteins [7, 8].

Recently, great efforts have been made to mine enzymes by culture-independent method of metagenomics using either sequence-based or function-based screening from environmental systems, e.g. soil, rumen, ocean, spring, gut, activated sludge, skin surface, compost, most of which are from prokaryotic microbes [9]. And, it is still very difficult to obtain enzymes of eukaryotic microorganisms from environments, due to difficulties in extracting polyadenylated messenger RNA (metatranscriptomics), and the lack of efficient and compatible hosts. At present time, only a few enzymes are reported to be identified by functional metatranscriptomics of eukaryotes from soil, rumen and the gut of the termite [10–13], or by sequence-based metatranscriptomics of eukaryotes from forest soil [14]. Same as soil, eukaryotic microbial communities also play pivotal roles in man-made environmental NF liquor starter, since NF liquor starter is mainly made from wheat [3, 8]. However, in a long time, the complicities in this man-made environment of NF liquor starter, e.g., the high content of starch and other polysaccharides, thousands of fermentation products at high temperature, limit the acquisition and characterization of active enzymes from this environment, and only crude enzymes are analyzed until now [7]. Recently, a breakthrough on NF liquor starter is made by metatranscriptomic analysis in our previous work, and it is found that fungi are the most abundant and active community members and total 932 carbohydrate-active enzymes are identified at the high temperature stage (N3) of 62 °C [3]. Moreover, many active enzymes with whole length gene sequences are identified. Therefore, metatranscriptomic analysis provides a direct and efficient sequence-based method to mine these fungal functional enzymes from NF liquor starter.

To verify the feasibility of this method, enzymes related to starch degradation are selected, since starch is the main composition of wheat material in NF liquor starter [8]. In the liquor starter, alpha-amylase makes the largest contribution to liquefaction of starch, and works efficiently with glucoamylase to produce fermentable reducing sugars [4, 8]. And thermostable α-amylases are more attractive in biotechnological processes, since they are stable over 60 °C and allow a higher operation temperature which result in higher reactivity, higher process yield, fewer contamination, and so on [15]. NF liquor starter is a promising resource for thermostable fungal α-amylase, since its making process is subjected to high temperature (N3) of 62 °C for 8 days. Moreover, based on previous metatranscriptomics analysis of NF liquor starter, enzymes related to starch metabolism, e.g., α-amylase, β-glucosidase and glucoamylase, showed relatively high expression level at high temperature stage (N3), and α-amylases exhibited the second highest RPKM (Reads Per Kilobase per Million) value up to 465.7, which stands for the second highest expressional enzymes in N3 [3]. Therefore, thermostable α-amylase is finally selected to be directly mined from NF liquor starter, which should have high efficiency in liquefaction of starch.

In the present study, we provided a direct and efficient way to mine enzymes from man-made environment of Chinese NF liquor starter, by which one fungal thermostable α-amylase was successfully obtained and characterized. This α-amylase was the highest expression enzymes related to starch hydrolysis at high temperature period (62 °C), and showed interesting characteristics, such as Ca^2+^-dependent on activity and thermostability, high stability at 60 °C, acidic pH optima at 5.0–5.5, the more efficient hydrolysis on amylopectin than on amylose, high activity on starches and tolerances of high substrates concentrations (15 mg/ml). This study would shell light on understanding the important role of this thermostable α-amylase in NF liquor starters and pave the way toward the optimization of liquor production and improvement of liquor quality. To the best of our knowledge, this is the first report of directly mining enzymes from NF liquor starter.

## Methods

### Materials

*Escherichia coli* DH5α strain (Sangon Biotech, Shanghai) was used for gene cloning and plasmid maintenance throughout the study, and *Pichia pastoris* X33 strain (Invitrogen, Carlsbad, CA, USA) was used for protein expression. The pGAPZαA vector (Invitrogen, Carlsbad, CA, USA) was used for gene cloning. Potato starch, wheat starch, corn starch, amylose (potato) and amylopectin (potato) were obtained from Sigma-Aldrich (St. Louis, MO). The Mix (Green) for PCR amplification was from TsingKe (Beijing, China). The QIAprep Spin Miniprep Kit and RNeasy^@^ Midi Kit were obtained from Qiagen (Valencia, CA). SMART^®^ cDNA Library Construction Kit, Advantage^®^ 2 PCR Kit and the Talon metal affinity resin were purchased from Clontech Laboratories, Inc. (Mountain View, CA). The Amicon^@^ ultra centrifugal filters were obtained from Millipore (Billerica, MA). All other reagents were purchased from general commercial suppliers and used without further purification.

### Liquor starter sampling, RNA extraction and cDNA library construction

NF liquor starter was sampled from a liquor fermentation factory in Yibing, Sichuan, China. As described in our previous work [3], sample N3 was collected after 9 days of liquor starter fermentation with temperature around 62 °C. Briefly, samples were frozen in liquid nitrogen immediately after obtained in factory, transferred to 50 ml RNase free tubes and kept in dry ice. Finally, all samples were transferred to Chengdu Biology Institute, Chinese Academy of Sciences and stored at − 80 °C for further research.

A previously published protocol [3] was used to extract total RNA from liquor starter with some modifications. In short, 1 g of liquor starter was homogenized into fine powder in a precooled mortar. Next, 4 ml borate buffer [200 mM sodium borate (pH 9.0), 30 mM ethyleneglycoltetraacetic acid (EGTA), 1% (w/v) sodium dodecyl sulfate (SDS), 4% (w/v) polyvinylpyrrolidone (PVP), and 0.5% (v/v) Nonidet-40 (NP-40), 10 mM β-mercaptoethanol and 0.03% (v/v) RNase inhibitor] and 280 µl of proteinase K (20 mg/ml) were mixed and incubated at room temperature for 2 min. The RNA of this crude lysate was then centrifuged, precipitated with 70% ethanol, and cleaned according to the RNeasy Midi Kit protocol. An extra treatment with DNase I (Fermentas, USA) on RNA mixture was also performed according to the manufacturer’s protocol.

Three microliters of total RNA (around 1 µg) was used as template for first-strand synthesis with MMLV Reverse Transcriptase, after which the cDNAs were amplified via 20 cycles of a long distance PCR (LD-PCR) using the SMART^®^ cDNA Library Construction Kit. The resulting environmental cDNAs were used as template to specifically amplify α-amylase gene sequence.

### Gene cloning, expression and protein purification

**Fig. 1:**
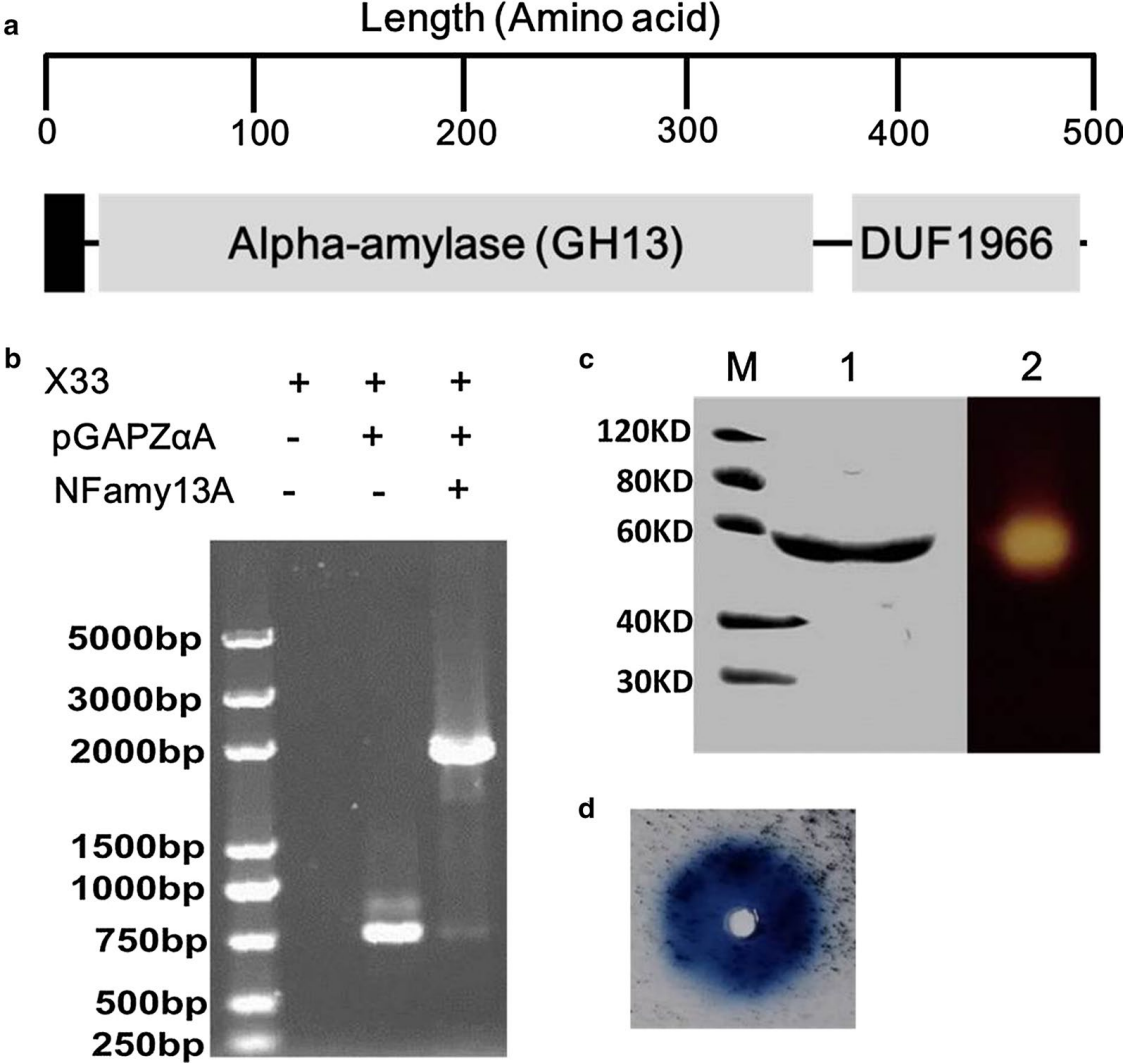
Schematic representation, genomic integration, Zymogram analyses and plate-based activity assays of NFAmy13A. **a** Schematic representation of NFAmy13A. **b** Verification of integration of NFAmy13A gene into genome of *P. pastoris* X33. **c** Zymogram analyse of amylase activities of native NFAmy13A. **d** Plate-based activity assay of native NFAmy13A. The signal peptide is represented by the filled rectangle. GH13: family 13 glycoside hydrolase domain. GUF1966: domain of unknown function 1966. Lane M, molecular mass markers; Lane 1, purified native NFAmy13A; Lane 2, zymogram of purified native NFAmy13A

The metatranscriptomics of liquor starter N3 was analyzed in our previous work. The gene product of ORF17558 was predictively composed of alpha-amylase domain and DUF1966 domain (DUF: domain of unknown function) (Fig. 1a), and it was designated NFAmy13A, since it is the first characterized alpha-amylase in Nong flavor liquor starter. The nucleotide sequence encoding the NFAmy13A was amplified with Mix (Green) using N3 cDNA as the template, with NFAf 5′GGTACCGCGACTCCGGATGAGTGGAAAGCTCAG3′ and NFAr5′ TCTAGACGCCGACGCACACAGACCACTCTTG3′ primers containing *Kpn*I and *Xba*I site, respectively. The PCR products and plasmid pGAPZaA (Invitrogen) were digested with *Kpn*I and *Xba*I (New England Biolabs). The resulting DNA fragments were ligated with T4 DNA ligase (New England Biolabs) and the ligated product was transformed into *E. coli* DH5a. The transformants were selected on low salt LB (10 g/l tryptone, 5 g/l NaCl and 5 g/l yeast extract) agar plate supplemented with 25 μg/ml zeocin (Sangon Biotech, Shanghai). Single colonies were inoculated into LB medium supplemented with same antibiotics, and cultured overnight. Plasmids were extracted (Qiagen mini-prep kit) from the cultures, and the inserts were verified by DNA sequencing (TsingKe, Chengdu, China).

The recombinant plasmid with correct insert (pGAPZaA/NFAmy13A) was linearized by Avr II and transformed into *P. pastoris* X33 using MicroPulser Electroporator (Bio-Rad Laboratories, Hercules, CA, USA) for gene expression. After 3 days incubation on YPD medium (10 g/l yeast extract, 20 g/l peptone and 20 g/l dextrose) supplemented with 1 M sorbitol and 100 μg/ml zeocin at 30 °C, single colonies were inoculated into 25 ml YPD medium with same concentrations of sorbitol and zeocin at 30 °C with vigorous shaking at 250 rpm for 2 days, and transformants with correct inserts were analyzed by PCR amplification with pGAP Forward (5′-GTCCCTATTTCAATCAATTGAA-3′) and 3′ AOX (5′-GCAAATGGCATTCTGACATCC-3′) primers according to Invitrogen protocol. The correct transformants were further verified by activity assays in supernatants after centrifugation at 6000*g* for 5 min at 4 °C.

The transformants with correct inserts were expressed in 500 ml YPD medium supplemented with same amount of sorbitol and zeocin at 30 °C and 250 rpm for 2 days. After centrifuge, supernatants were heated twice at 55 °C for 15 min since proteins from N3 were expected to be thermostable, and the precipitated host proteins were pelleted by centrifugation at 12,000×*g* for 10 min at 4 °C. Then, supernatants were filtered through 0.45 μm MF-Millipore membrane and adjusted to Tris–HCl buffer (pH 7.5, 50 mM Tris–HCl, 300 mM NaCl). The resulting supernatants were applied to the talon metal affinity resin and the recombinant proteins with N-terminal 6-Histidine-tag were purified according to the manufacturer’s instruction (Clontech). The bound proteins were eluted with the elution buffer (150 mM imidazole, 50 mM Tris–HCl, 300 mM NaCl, pH 7.5), and the fractions were analyzed by sodium dodecyl sulfate–polyacrylamide gel electrophoresis (SDS-PAGE). The fractions containing the purified NFAmy13A proteins were concentrated, the buffer of which was changed to a protein storage buffer (50 mM Tris–HCl, 150 mM NaCl, pH 7.5) with Amicon Ultra-10 centrifugal filters. The protein concentrations were measured using a NanoDrop 2000c (Thermo Fisher Scientific Inc., Waltham, MA) and the Beer-Lambert law with the extinction coefficients of 91,010/M/cm for NFAmy13A.

### Zymogram analysis and plate-based activity assay of NFAmy13A

For zymogram analysis, around 20 μg purified NFAmy13A was loaded onto 10% native-PAGE gel (without SDS). After electrophoresis, the gel was washed with 50 mM citrate buffer (pH 5.5) for three times. Then the gel was overlaid on a sheet containing 1% soluble starch with 1.5% agar and 0.01% Trypan Blue in 50 mM citrate buffer (pH 5.5) for 1 h at 60 °C. Finally, amylase activity was visualized as a clear zone by staining this sheet with Lugol’s iodine solution.

For plate-based activity assay, around 20 μg purified NFAmy13A was added onto the center hole of plate containing 0.1% AZCL-amylose (Megazyme) with 1% agarose in 50 mM citrate buffer (pH 5.5), and the plate was kept at 60 °C for 2 h. Then the amylase activity was visualized with blue zone around the hole.

### Enzyme assays

The optimal pH of NFAmy13A was determined by incubating 40 nM NFAmy13A with 5 mg/ml potato starch between pH 4.0 and 9.0 (50 mM citrate buffer for pH 4.0–6.0, 50 mM sodium phosphate buffer for pH 6.0–8.0, 50 mM Tris–HCl for pH 8.0–9.0) at 55 °C for 30 min. The reducing sugars released were measured using the *para*-hydroxybenzoic acid hydrazide (*p*HBAH, Sigma-Aldrich, St. Louis, MO) assay [16] with glucose as the standard. Meanwhile, the optimal temperature was determined by incubation of 40 nM NFAmy13A and 5 mg/ml potato starch at its optimal pH at different temperatures ranging from 30 to 80 °C for 30 min.

The specific activity of NFAmy13A was determined by incubating 20 nM enzymes with 5 mg/ml potato starch at 60 °C and pH 5.5 for different time intervals, within the range where the slopes of released glucose equivalents versus time were linear. One unit of alpha-amylase activity was defined as the quantity of enzyme capable of releasing 1 μmol glucose equivalent per minute under its optimized conditions. The digestion of various substrates was also characterized by incubating 100 nM NFAmy13A with 5 mg/ml substrates at 60 °C and pH 5.5 for 30 min.

### Thermostability assay

To determine the thermostability of NFAmy13A, enzymes (80 nM) were incubated in a citrate buffer (pH 5.5) at 50, 60 and 70 °C in THERMO SHAKER. Samples were taken out at different time points and placed on ice. The residual enzymatic activity was determined by incubating 40 nM enzymes with 5 mg/ml amylopectin at 60 °C in a citrate buffer (pH 5.5) for 30 min.

### Effect of metal salts and chemical additives on amylase activity

The effect of various metal salts was studied by adding 1 and 10 mM metal salts and additives to the reaction mixture of 100 nM enzymes with 5 mg/ml amylopectin under standard assay. Relative activities were calculated as a percentage of the activity of the unadded control as 100%.

### Kinetics hydrolysis of NFAmy13A on starch substrates

The kinetics studies of NFAmy13A were determined by incubating purified NFAmy13A with different concentrations of starch substrates, i.e., amylopectin, amylose, potato starch, corn starch or wheat starch, ranging from 0.1 to 18 mg/ml. The reducing ends were measured after incubation under normal condition for 30 min in the presence of 10 mM CaCl_2_, and the velocities were kept in a constant scope under every condition by using low concentration of 20 nM enzyme.

### Application of NFAmy13A in the hydrolysis of raw potato starch substrates

To measure the effect of concentrations of starch substrates, i.e., potato starch, corn starch or wheat starch, their concentrations were varied from 1 to 18 mg/ml in citrate buffer (pH 5.5) containing high concentration of 1 μM enzymes and 10 mM CaCl_2_. After incubation at 60 °C for 20 h, the concentration of reducing ends was estimated using the *p*HBAH assay as described above. Then, the end products was analyzed by thin-layer chromatography (TLC) with glucose (M1) and maltose-oligosaccharides (M2–M6) as standards and *n*-butanol-acetic acid-H_2_O (10:5:1, vol/vol/vol) as developing mobile phase. The sugars were visualized by spraying with a 1:1 (vol/vol) mixture of methanolic orcinol (0.2% wt/vol) and sulfuric acid (20% vol/vol) followed by heating at 100 °C for 5 min [17].

### Nucleotide sequence accession numbers

The nucleotide sequence of *NFAmy13A* was deposited at the GenBank database with accession number MF765801.

## Results

### RNA extraction and cDNA library construction

Total RNA was extracted from 1 g of liquor starter sample N3 and approximately 15 μg RNA was obtained with high purity. Full-length cDNA was synthesized by the initial reverse transcription and enriched by subsequent LD PCR. When analyzed by agarose gel, environmental double strands cDNA appeared as a strong smear ranging from 100 bp to 4 kb, and it was adequate for the subsequent specific amplification.

### Cloning, expression, and purification of NFAmy13A

ORF17588 was identified to predictively encode an alpha-amylase, which was designated as NFAmy13A since it was the first characterized alpha-amylase in NF liquor starter. Based on our previous metatranscriptomics analyses of NF liquor starter, NFAmy13A was the only alpha-amylase in the top 20 highest expression level of glycoside hydrolases in N3 (Additional file 1: Table S1) with RPKM value of 293.5, and accounted for around 63% of total alpha-amylases in N3 (Additional file 1: Table S2), thus being the highest expression level of alpha-amylases in starch degradation of N3. In addition, NFAmy13A also showed active expression with RPKM value of 30.9 in mature stage of NF liquor starter according to our previous metatranscriptomic analysis (Additional file 1: Table S2). The wheat material of NF liquor starter is rich in starch. Therefore, NFAmy13A could play a pivotal role in liquefaction of NF liquor starter at high temperature stage and mature stage, and it was characterized in present work.

The amino acid sequence of NFAmy13A was analyzed by the Pfam server (http://pfam.sanger.ac.uk/), and it had alpha-amylase domain and DUF1966 domain (Fig. 1a). The product of NFAmy13A gene had a signal peptide when predicted on SignalP 4.1 Server (http://www.cbs.dtu.dk/services/SignalP/) (Fig. 1a). The NFAmy13A gene sequence (1431 bp), without signal peptide sequence, was successfully obtained by specific amplification with primers NFAf/NFAr from ds cDNA of N3, and one colony with correct gene integration into genome of *P. pastoris* X33 was identified by PCR with primers pGAP Forward/3′ AOX from three colonies (Fig. 1b). This colony showed the highest activity of 28.3 U/ml when cultured for 72 h. NFAmy13A was purified to near homogeneity, and the apparent molecular masses of the purified recombinant NFAmy13A corresponded well with its calculated values (56.1 KD) (Fig. 1c). Obviously, purified NFAmy13A showed strong amylase activity in degrading starch in a native-PAGE gel (Fig. 1c) and an agarose plate (Fig. 1d), when analyzed with zymogram method and plate-based activity assays, respectively.

### Characterization of NFamy13A

**Fig. 2:**
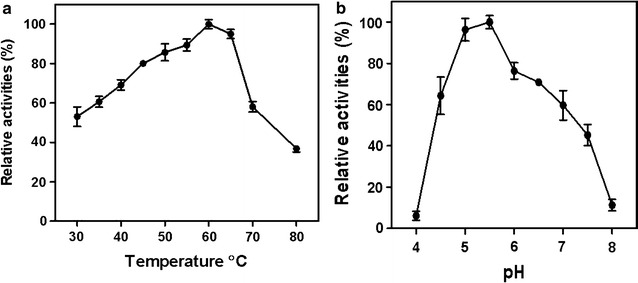
The effects of pH and temperature on the activity of the purified NFAmy13A. **a** The effect of temperature on the activity of NFAmy13A was determined by incubation of 40 nM NFAmy13A and 5 mg/ml potato starch at 5.5 at temperatures ranging from 30 to 80 °C. **b** The effect of pH on the activity of NFAmy13A was determined by incubating 40 nM NFAmy13A with 5 mg/ml potato starch between pH 4.0 and 9.0 (50 mM citrate buffer for pH 4.0–6.0, 50 mM sodium phosphate buffer for pH 6.0–8.0, 50 mM Tris–HCl for pH 8.0–9.0) at 55 °C. The concentration of reducing ends was estimated using the *p*HBAH assay. All values are expressed as percentages of the highest activity at one detected conditions

NFAmy13A from the highest temperature (62 °C) stage N3, showed optimal activity at 60 °C and more than 80% of activity remaining between 45 and 65 °C (Fig. 2a), to some extent, which could support the efficient hydrolysis of starch by NFAmy13A at high temperature of 62 °C in NF liquor starter. Maximum α-amylase activity was observed at pH 5–5.5 for NFAmy13A, and there were still 50% of activity remaining between pH 4.5 and 7.0 (Fig. 2b). Therefore, standard assays were performed at pH 5.5 and 60 °C for 30 min throughout this work, except for special mentioned.

Furthermore, according to the hydrolysis result (Table 1), NFamy13A had higher activity on amylopectin than on amylose, and the activities on other starch substrates, i.e., potato starch, wheat starch, corn starch, maltodextrin, and white dextrin, were between those on amylopectin and amylose. Meanwhile, NFAmy13A also showed some activities on substrates of pullulan and glycogen. Therefore, the specificity activity was determined as 200.4 U/mg when using amylopectin as substrate.

**Table 1:** The hydrolysis of various substrates catalyzed by NFAmy13A

Substrate	Main linkage/monomer	Relative activity (%)
Amylopectin (potato)	α(1 → 4)–α(1 → 6) glucose	100.0 ± 4.9
Amylose (potato)	α(1 → 4) glucose	15.5 ± 1.1
Potato starch	α(1 → 4)–α(1 → 6) glucose	62.6 ± 9.4
Wheat starch	α(1 → 4)–α(1 → 6) glucose	21.8 ± 1.8
Corn starch	α(1 → 4)–α(1 → 6) glucose	30.7 ± 1.6
Maltodextrin	α(1 → 4) glucose	18.9 ± 1.9
White dextrin (corn)	α(1 → 4)–α(1 → 6) glucose	66.1 ± 4.8
Pullulan	α(1 → 6)–α(1 → 4) glucose	2.3 ± 1.5
Dextran T500	α(1 → 6)–α(1 → 3) glucose	0
Glycogen	α(1 → 4)–α(1 → 6) glucose	11.2 ± 0.6
CMC	β(1 → 4) glucose	0
Cellulose	β(1 → 4) glucose	0

The activity of NFAmy13A is influenced by the presence of external factors such as cations and additives. As shown in Table 2, at lower concentration of 1 mM, all of metal salts and additives did not show any effects on the activity of NFAmy13A, except Hg^2+^, Co^2+^ and SDS, which showed some inhibitions. Meanwhile, at higher concentration of 10 mM, Ca^2+^ significantly stimulated the activity of NFAmy13A with 47% enhancement, and Mg^2+^ slightly stimulated its activity, which might suggest the enzyme needs a cofactor for its maximum activity. Among the cations and additives tested, 10 mM Hg^2+^ largely inhibited amylase activity. Under the same concentrations of 10 mM, Co^2+^ and SDS inhibited 50 and 40% of amylase activity, respectively; Fe^2+^, Ni^2+^, Cu^2+^ and EDTA between 10 and 20%, while other cautions had little effect on alpha-amylase activity.

**Table 2:** Effects of different metal salts and chemical additives on activity of NFAmy13A

Additives	Relative activity (%)	
	1 mM	10 mM
α-Amylase (no additives)	100.0 ± 2.5	100.0 ± 1.1
CaCl_2_	98.1 ± 1.1	147.5 ± 5.1
AlCl_3_	96.4 ± 5.0	98.9 ± 5.1
BaCl_2_	94.3 ± 3.2	90.8 ± 2.7
CoCl_2_	64.6 ± 0.8	47.5 ± 3.9
FeCl_3_	93.2 ± 1.7	81.1 ± 5.9
CuCl_2_	95.0 ± 0.6	83.9 ± 0.0
KCl	97.7 ± 0.9	95.7 ± 0.5
MgSO_4_	95.5 ± 2.8	115.8 ± 3.2
ZnSO_4_	97.2 ± 0.4	90.9 ± 1.4
NiSO_4_	95.0 ± 2.6	84.2 ± 5.4
MnSO_4_	94.4 ± 2.0	92.3 ± 1.4
HgCl_2_	49.0 ± 4.4	2.2 ± 0.6
EDTA	97.8 ± 0.8	87.2 ± 3.9
SDS	80.0 ± 3.7	61.7 ± 6.7

**Fig. 3:**
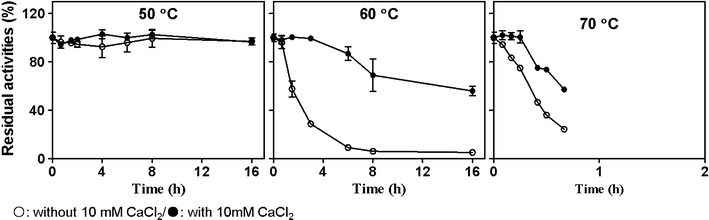
The effect of temperature on the stability of the NFAmy13A. The enzyme (80 nM) was incubated in a citrate buffer (pH 5.5) at 50, 60 and 70 °C for different times in the presence (filled circle) or absence (circle) of 10 mM CaCl_2_. The residual enzymatic activity was determined by incubating 40 nM enzymes with 5 mg/ml amylopectin at 60 °C in a citrate buffer (pH 5.5) for 30 min. All values are expressed as percentages of the activity of untreated enzyme

The temperature of NF liquor starter at N3 stage is 62 °C, thus, the thermostable amylases are of great importance for starch hydrolysis. The thermal stability of NFAmy13A was detected at 50, 60 and 70 °C in the presence and absence of 10 mM CaCl_2_, and the thermostability was positively affected by 10 mM Ca^2+^ (Fig. 3). At low temperature of 50 °C, NFAmy13A was very stable with almost 100% residual activities after incubated for 16 h. Interesting, at 60 °C, the activity of NFAmy13A dropped to 28% after 3 h, while in the presence of CaCl_2_ enzyme retained 100% activity after the same incubation time. Moreover, at 70 °C, the activity dropped to 24% after 40 min, and enzyme kept 57% activity after the same incubation time. The half-lives at 60 and 70 °C were improved significantly from 1.5 and 0.4 h to 16 and 0.7 h, respectively, in the presence of 10 mM CaCl_2_.

**Fig. 4:**
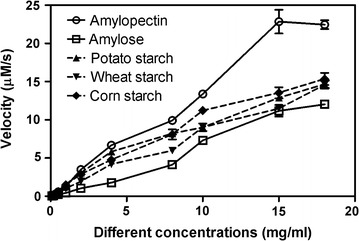
Kinetics hydrolysis of NFAmy13A on starch substrates. The purified NFAmy13A (20 nM) was incubated with different concentrations (0.1–18 mg/ml) of starch substrates, i.e., amylopectin, amylose, potato starch, corn starch or wheat starch, for 30 min in the presence of 10 mM CaCl_2_

As shown in Fig. 4, the kinetics hydrolysis of several starch substrates by NFAmy13A did not fit to a Michaelis–Menten equation, and velocities reached the highest point on substrates amylopectin and amylose when the concentration was 15 mg/ml. NFAmy13A showed the highest values of velocity on amylopectin and the lowest values on amylose, while the velocities on potato starch, wheat starch and corn starch were in middle at all detecting concentrations of substrates. Those results were similar as observed in Table 2.

### Application of NFAmy13A in the hydrolysis of starch substrates

**Fig. 5:**
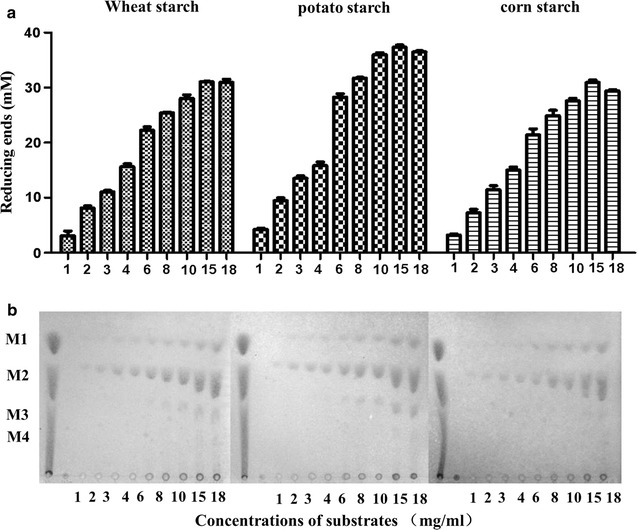
Hydrolysis of increasing amounts of starch substrates by NFAmy13A. The products were analyzed by both reducing sugar assay (**a**) and TLC analysis (**b**). Different concentrations (1–18 mg/ml) of potato starch, corn starch or wheat starch were incubated with 1 μM enzymes in the presence of 10 mM CaCl_2_ at pH 5.5 and 60 °C for 20 h. M1, glucose; M2, maltose; M3, maltotriose; M4, maltotetraose

To mimic the hydrolysis of wheat starch in liquor starter, starch substrates were completely dissolved in warm buffer (60–80 °C), and varied concentrations (from 1 to 18 mg/ml) of substrates were hydrolyzed with a constant enzyme dose (1 μM). As shown in Fig. 5a, at the same enzyme concentration, increasing any of the three substrates led to release of more products. The releasing reducing sugars reached the highest values at high concentration of 15 mg/ml for all three substrates, and more reducing sugars were released from potato starch than wheat starch and corn starch. The main end products for all three substrates were maltose, and small amounts of glucose were released throughout most of the conditions (Fig. 5b). Minor maltotriose was also clearly detected at concentration of 15 and 18 mg/ml substrates. More glucose and maltose-oligosaccharides were detected at higher concentration of substrates, and more were released from potato starch and wheat starch than from corn starch.

## Discussion

NF liquor starter is enriched with special and stable microbial communities after several 1000 years’ evolution, and plenty of active enzymes are found with efficient capacity in degrading carbohydrates and protein. However, in a pretty long time, few active enzymes were mined and characterized from NF liquor starter by traditional culture based methods. Recently, our previous metatranscriptomic study on NF liquor starter might provide a direct and easy sequence-based method to mine those functional enzymes and understand their roles in NF liquor brewing. Among these enzymes, α-amylases play a pivotal role in the liquefaction of starch, and thermostable α-amylases gains more attentions due to their potential applications at a high operation temperature (> 60 °C) compared to mesophilic α-amylases. Therefore, this study was performed to verify the feasibility of directly mining enzymes from NF liquor starter, and a thermostable α-amylase (NFAmy13A) was successfully obtained from high temperature period (N3).

**Fig. 6:**
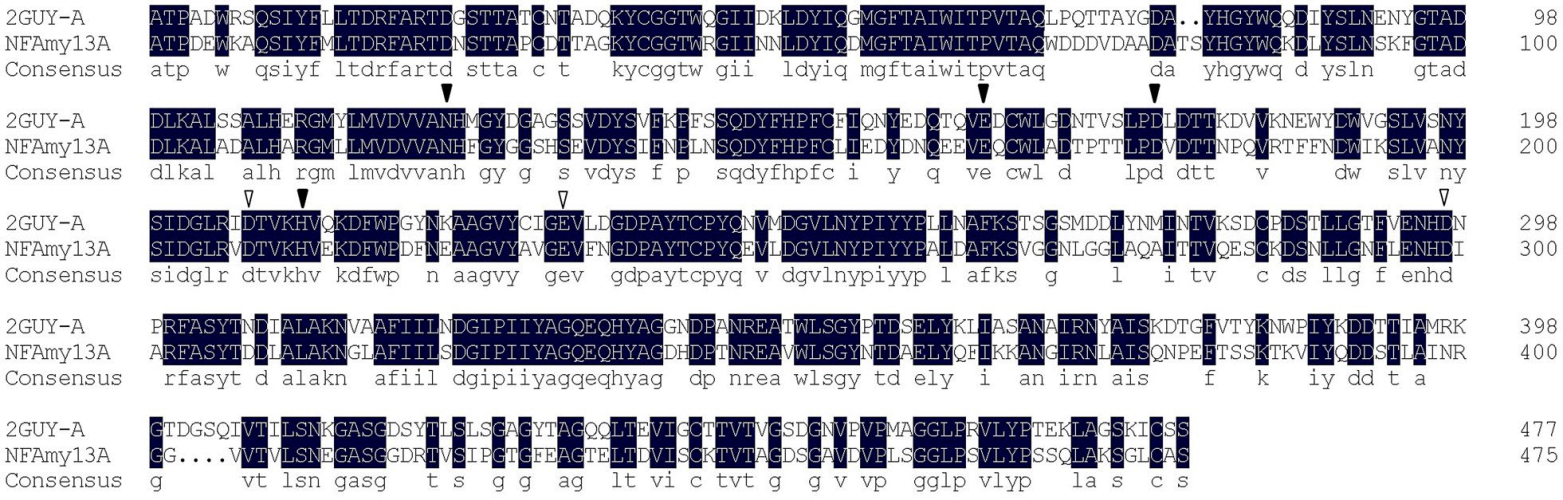
Protein sequences alignment between NFAmy13A and α-amylase (crystal structure: 2GUY-A) from *Aspergillus niger*. The conserved catalytic sites were highlighted with ‘inverted triangle’, and the conserved Ca^2+^-binding sites were highlighted with ‘filled inverted triangle’

Protein sequence analysis showed that NFAmy13A had a signal peptide and, without signal peptide, it shared the highest sequence identity of 64% with α-amylase (crystal structure: 2GUY-A) from *Aspergillus niger*, thus possibly originating from a fungal genus. As we know, fungal α-amylases are more preferred for starch hydrolysis in traditional food industries, e.g., brewing (Chinese liquor, wheat wine, soy sauce and vinegar), baking and sweeteners, and pharmaceutical industries due to their nontoxic characteristics [8, 18, 19]. In addition, three catalytic sites (Asp206, Glu230, Asp297) in 2GUY-A were also conserved in NFAmy13A (Asp208, Glu232, Asp299) (Fig. 6), so NFAmy13A might have same hydrolysis reaction [20]. Moreover, most of the reported fungal α-amylases were native proteins (Table 3), and only a few were heterologously expressed in *E. coli* [21], *Aspergillus oryzae* [22], *P. pastoris* [23–26], or *Saccharomyces cerevisiae* [27].

**Table 3:** Comparison of enzymatic characteristics among NFAmy13A and previously reported fungal α-amylases

Source fungi	Specificity activity (U/mg)	Optimal pH	Optimal temperature (°C)	Thermostability	Inhibitor	Activator	Expression host	References
Liquor starter (Fungus)	200.4	5–5.5	60	t_1/2_: 1.5 h at 60 °C, 0.4 h at 70 °C; in the presence of 0.01 M CaCl_2_, t_1/2_: 16 h at 60 °C, 0.7 h at 70 °C	Co^2+^, Hg^2+^, SDS	Ca^2+^, Mg^2+^	*Pichia pastoris*	This study
*Aspergillus awamori* KT-11	78.9	4	50	ND	Zn^2+^, Cu^2+^, Hg^2+^	Fe^2+^	Native	[38]
*Aspergillus flavus* var. columnaris	ND	5	50	96% loss of activity in absence of Ca^2+^, 10% loss of activity in presence of 0.01 M CaCl_2_ after incubation at 60 °C for 10 min	None	Ca^2+^	Native	[43]
*Aspergillus fischerianus*	2314.1	4.5	50	t_1/2_: 17 min at 45 °C	Cu^2+^	Co^2+^, Mn^2+^, Fe^3+^	*Pichia pastoris*	[23]
*Aspergillus niger* CBS 513.88	2.2	5.5–6.5	NA	Lost rapidly at above 35 °C for 10 min	Na^+^	None	*Escherichia coli* BL21	[21]
*Aspergillus niger* JGI 24	75	9	30	Around 20% loss of activity after incubation at 30 °C for 50 min	Na^+^, Mg^2+^	Ca^2+^,Co^2+^	Native	[32]
*Aspergillus niveus*	ND	6.0	65	t_1/2_: 20 min at 70 °C	Hg^2+^, Ag^+^, Fe^3+^	Ca^2+^, NH_4_^+^, Na^+^, K^+^, Mn^2+^, Mg^2+^	Native	[33]
*Aspergillus oryzae* ATCC 76080	410	4–5	50	Lost 80% activity after incubation at 50 °C for 30 min	Hg^2+^, DNFB, NBSI	None	Native	[39]
*Aspergillus oryzae* EMS-18	1987.7	5.0	40	ND	Mg^2+^, Mn^2+^, Na^+^, Zn^2+^, Ni^2+^, Fe^2+^, Cu^2+^, Co^2+^ and Ba^2+^	Ca^2+^	Native	[44]
*Aspergillus oryzae* S2	5089.3, 6686.3	5.6	50	t_1/2_: 60 min at 50 °C	Mg^2+^, Fe^2+^, Ba^2+^, Cu^2+^, Co^2+^, Mo^2+^, and Mn^2+^	Ca^2+^	Native	[48]
*Aspergillus tamarii*	778.3	4.5–6.5	50–55	t_1/2_: 30 min at 70 °C	Pb^2+^, Cu^2+^, Ag^+^, Fe^3+^	None	Native	[53]
*Geomyces pannorum*	12,800	5.0	40	40% loss of activity after incubation at 50 °C for 10 min	Ni^2+^, EDTA, Methanol, Teween-20/80	Ca^2+^, Mg^2+^	*Aspergillus oryzae*	[22]
*Malbranchea cinnamomea* S168 amylose	2240.7	6.5	65	Around 50% loss of activity after incubation at 60 °C for 30 min	Hg^2+^, Cr^3+^	Mn^2+^, Co^2+^	Native	[40]
*Paecilomyces variotii*	612.5	4	60	t_1/2_: 53 min at 60 °C	Cu^2+^, Al^3+^Fe^3+^, EDTA, K^+^, Pb^2+^, Zn^2+^	Ca^2+^, Co^2+^, Mn^2+^	Native	[34]
*Pichia burtonii*	ND	4	40	20% loss of activity after incubation at 50 °C for 30 min	Cd^2+^, Cu^2+^, Hg^2+^, Al^3+^, and Zn^2+^	None	Native	[41]
*Preussia minima* EL-14	138	9	25	ND	Na^+^, Mg^2+^	Mn^2+^, Ca^2+^	Native	[31]
*Rhizopus oryzae*	1123	4–6	60	50% loss of activity after incubation at 60 °C for 5 min	Cu^2+^, Fe^2+^,	Independence of Ca^2+^	*Pichia pastoris*	[24]
*Rhizomucor pusillus* GX-3	20,732	5	70	t_1/2_: 75 min at 60 °C	ND	ND	*Pichia pastoris*	[25]
*Saccharomycopsis fibuligera* KZ, amylose	120–125	5–6	40–50	ND	ND	ND	*Saccharomyces cerevisiae*	[27]
*Sclerotinia sclerotiorum*	ND	4	55	Around 80% loss of activity after incubation at 55 °C for 30 min	Cu^2+^, Mn^2+^, Ba^2+^, Fe^2+^	Weakly stimulated by Ca^2+^	Native	[45]
*Scytalidium thermophilum*	5.6	6.0	60	t_1/2_: 12 min at 60 °C	Cu^2+^, Hg^2+^	Independence of Ca^2+^	Native	[42]
*Streptomyces megasporus*	847.3	6	60	t_1/2_: 2 h at 70 °C	Ag^+^, Ba^2+^, Co^2+^, Cu^2+^, EDTA, Hg^2+^, Mg^2+^, Pb^2+^, Zn^2+^	None	Native	[35]
*Thermobifida fusca* NTU22	ND	7	60	25% loss of activity after incubation at 60 °C for 3 h	ND	ND	*Pichia pastoris*	[26]
*Thermomyces lanuginosus*	349	5	70	32 and 94% loss of activity after incubation at 50 °C for 2 and 18 h	Cu^2+^, FE^3+^	Ba^2+^, Ca^2+^, Cd^2+^, Mg^2+^, Mn^2+^	Native	[46]
*Thermomyces lanuginosus*	ND	5.6	65	At 65 °C (t_1/2_: 0.6 h) the enzyme is nearly 8 times more stable in presence of Ca^2+^	None	Ca^2+^	Native	[47]
*Thermomyces lanuginosus*	ND	5.2	60	t_1/2_: 140 min at 60 °C, 10 min at 70 °C	ND	ND	Native	[36]
*Talaromyces pinophilus* 1–95	673.1	4.0–5.0	55	60% loss of activity after incubation at 55 °C for 1 h	Ag^+^, Cu^2+^, Mn^2+^	Co^2+^, Fe^2+^, Fe^3+^	Native	[49]

ND, not determined

Similar to most of fungal α-amylases, NFAmy13A had acidic pH optima at < 5.5 (Table 3), which is preferred in starch industries [28–30]. On the contrary, some fungal α-amylases showed high pH optima at > 7.0 [26, 31, 32], which could be used in detergent industry. As shown in Table 3, most of fungal α-amylases are unable to work at temperatures more than 50–60 °C. Meanwhile, NFAmy13A had an optimum temperature at 60 °C and was stable with half-life of 1.5 h at 60 °C. Similar or higher thermostabilities were also found in a few fungal amylases from *Aspergillus niveus* [33], *Paecilomyces variotii* [34], *Rhizomucor pusillus* [25], *Streptomyces megasporus* [35] and *Thermomyces lanuginosus* [36]. Thermostable fungal α-amylases are mostly used in the starch hydrolysis of baking and brewing industries [8, 37].

Among the cation and additives tested, 10 mM Hg^2+^ largely inhibited NFAmy13A activity, which has been well observed on some fungal alpha-amylases [33, 35, 38–42]. The stimulation of Ca^2+^ was also found in other fungal alpha-amylases [22, 31–34, 43–48]. The ‘high-affinity’ Ca^2+^-binding sites (Asn121, Glu162, Asp175, His210) in 2GUY-A were highly conserved in NFAmy13A (Asn123, Glu164, Asp177, His212) (Fig. 6), indicating that NFAmy13A was a metalloenzyme and strongly bound Ca^2+^. Similar as some α-amylases reported previously [22, 30, 33, 49], NFAmy13A was insensitive to EDTA, and one of the possible reason might be that the affinity of calcium to NFAmy13A was stronger than that of EDTA. Moreover, the stabilizing effect of Ca^2+^ on thermostability was observed in NFAmy13A, which might be explained due to the formation of a calcium–sodium–calcium metal triad in the main Ca^2+^-binding site of the enzyme [50], and has also been found in some fungal alpha-amylases [43, 47].

As shown in Table 1 and Fig. 4, NFAmy13A showed the maximum activity on amylopectin, followed by starch, amylose, glycogen, and pullulan at all detected concentrations, and its specificity activity on amylopectin was 200.4 U/mg, which was within the range values of the reported fungal α-amylases (Table 3). In general, amylopectin has α-1,4 glycosidic bond in the main chain and α-1,6 glycosidic bond in the branching chain, resulting in many end points onto which enzymes can easily attach. Meanwhile, amylose only has α-1,4 glycosidic bond in the main chain and forms tightly packed helical structure. Possibly, their structural differences were one of the reasons that NFAmy13A was more efficient to degrade amylopectin than amylose. This preference of NFAmy13A on amylopectin further verified its important role in NF liquor starter, since wheat material of NF liquor starter has higher amylopectin content of its starch than its amylose content [51]. Moreover, this preference of NFAmy13A potentially enhance its pivotal role in the following stage of alcohol fermentation, because the main material of alcohol fermentation is sorghum [8] and sorghum also has higher amylopectin content [52]. Similar preference between amylopectin and amylose was also found in other fungus α-amylase [22, 25]. Whereas, some fungal amylases showed higher activity on amylose than on amylopectin [21, 40, 49, 53]. Similar as amylopectin, glycogen is also composed with main chain of α-1,4 glycosidic bond and side chain of α-1,6 glycosidic bond, but being more branched and compact. This might be the reason that NFAmy13A could catalyze glycogen effectively and the degradation efficiency on glycogen is lower than that on amylopectin, which was also observed in other fungal α-amylases [22, 25, 48]. In addition, NFAmy13A exhibited very low activity toward pullulan, which perhaps resulted from the resistant structure that maltotriose (α-1,4 glycosidic bond) unites are connected to each other by α-1,6 glycosidic bond in pullulan. Most other fungal α-amylases also showed weak activity toward pullulan [22, 48, 49, 53], except for fungal α-amylase from *Malbranchea cinnamomea*, which showed high activity on pullulan [40].

NFAmy13A showed high activity toward soluble starches from corn, wheat, and potato, and worked efficiently at high concentrations up to 15 mg/ml. Its end products were maltose, glucose and minor maltotriose. Among them, main products of maltose and glucose can be easily uptake by yeast in the following alcohol fermentation stage, which further verified its pivotal role in the liquor fermentation. Therefore, as shown in Table 3, compared with other fungal α-amylases, NFAmy13A showed some advantageous characteristics in hydrolyzing starch substrates, e.g., high thermostability at 60 °C, acidic pH optima at 5.0–5.5, Ca^2+^-dependent, high activity on wheat starch, the more efficient hydrolysis on amylopectin than on amylose, and tolerances at high substrates concentrations (15 mg/ml), which to some extent proved previous finding that NFAmy13A was the highest expressed α-amylase at 62 °C and relatively high expressed α-amylase at mature stage in NF liquor starter, thus being the largest contribution in liquefying starch into glucose and maltose at solid state at high temperature stage and mature stage [3]. Moreover, as we know, corn and wheat, are also widely used for fuel ethanol production [54], and high activity toward corn and wheat highlights the potential applications of NFAmy13A in ethanol industrial. In addition, taking into account the safe advantage of fungal α-amylase, NFAmy13A was a good candidate in starch hydrolysis in food and pharmaceutical industries.

## Conclusions

A direct and efficient sequence-based method of metatranscriptomics is provided to mine active enzymes from NF liquor starter in present work. And by this way, a thermostable fungal α-amylase (NFAmy13A) gene is successfully obtained from environmental cDNA library of NF liquor starter, and expressed in *P. pastoris*. The recombinant enzyme of NFAmy13A has optimal pH of 5.0–5.5 and optimal temperature as high as 60 °C. When compared with other fungal α-amylases, NFAmy13A shows interesting characteristics, such as high thermostability, Ca^2+^-dependent on activity and thermostability, high activity on amylopectin, starches (from corn, wheat, and potato), amylose and glycogen, the more efficient hydrolysis on amylopectin than on amylose, and tolerances of high substrates concentrations. Therefore, NFAmy13A not only plays pivotal role in the solid-state SSF of starch liquefaction in NF liquor brewing, but also has potential application in the solid-state SSF process of other food industries, and pharmaceutical industries. Successfully mining α-amylase (NFAmy13A) from NF liquor starter will stimulate the characterization of more enzymes related to polysaccharides degradation in future, such as starch, cellulose, and hemicellulose. Their synergistic roles in degrading polysaccharides in liquor starter will also be studied, thus paving the way toward the optimization of liquor production and improvement of liquor quality, and stimulating potential application as safe additive for other industrial applications, such as bioenergy production, food or feed preservation and high valued-added products.

## Additional file

**Additional file 1.** Additional tables.
