# Supplementary material for: Directly mining a fungal thermostable α-amylase from Chinese Nong-flavor liquor starter
Source: Microb Cell Fact. 2018 Feb 22;17:30. doi: 10.1186/s12934-018-0878-y (PMC5822527; doi:10.1186/s12934-018-0878-y)
Supplement: Supplementary file 1 — Additional file 1. Additional tables. [file 12934_2018_878_MOESM1_ESM.docx]

Additional file

Directly mining a fungal thermostable α-amylase from Chinese Nong-flavor liquor starter

Zhuolin Yi^1,2,3^, Yang Fang^2,3^, Kaize He^2,3^, Dayu Liu^1^, Huibo Luo^4^, Dong Zhao^5^, Hui He^6^, Yanling Jin^2,3*^, Hai Zhao^1,2,3*^

*** Correspondence:**Hai Zhao (email: [zhaohai@cib.ac.cn](mailto:zhaohai@cib.ac.cn)) and Yanling Jin (email: jinyl@cib.ac.cn), Environmental Microbiology Key Laboratory, Chengdu Institute of Biology, CAS, No.9 Section 4, Renmin Nan Road, Chengdu, 610041, Sichuan, P.R. China. Phone: +86 28 82890725; Fax: +86 28 82890733;

Additional table 1. The top 20 highest expression of genes related to carbohydrate-active enzymes at the highest temperature stage (N3) of NF liquor starter

| GeneID | RPKM | Class | Class_description |
| --- | --- | --- | --- |
| 31847 | 655.5 | GH31 | alpha-glucosidase, alpha-xylosidase, alpha-glucan lyase |
| 17772 | 568.6 | GT30 | α-3-deoxy-D-manno-octulosonic-acid (KDO) transferase |
| 11929 | 549.0 | GH0 | Glycoside hydrolases not yet assigned to a family. |
| 8756 | 465.3 | GH3 | beta-glucosidase, 1,4-beta-xylosidase,alpha-L-arabinofuranosidase |
| 17445 | 381.2 | CBM18/GH16 | endo-1,3-beta-glucanase,xyloglucanase,endo-beta-1,3-galactanase |
| 44876 | 341.2 | GH11 | xylanase |
| 38358 | 330.3 | GH3 | beta-glucosidase, xylan 1,4-beta-xylosidase, alpha-L-arabinofuranosidase |
| 24092 | 327.5 | CE0 | Carbohydrate esterases not yet assigned to a family |
| 12023 | 316.1 | GH16 | endo-1,3-beta-glucanase, xyloglucanase, endo-beta-1,3-galactanase |
| 3131 | 305.4 | GH61（AA9） | copper-dependent polysaccharide monooxygenases |
| 23023 | 304.2 | GT2 | cellulose synthase,chitin synthase |
| 17558  (NFAmy13A) | 293.5 | GH13 | alpha-amylase,pullulanase |
| 22702 | 290.3 | GH16 | endo-1,3-beta-glucanase,xyloglucanase,endo-beta-1,3-galactanase |
| 26838 | 288.9 | GT2 | cellulose synthase, chitin synthase |
| 21154 | 286.9 | CBM18/GH16 | endo-1,3-beta-glucanase,xyloglucanase,endo-beta-1,3-galactanase |
| 30117 | 264.9 | GT41 | UDP-GlcNAc: peptide beta;-N-acetylglucosaminyltransferase |
| 11999 | 257.4 | CBM43/GH72 | beta-1,3-glucanosyltransglycosylase |
| 27126 | 242.5 | GH27 | alpha-galactosidase,alpha-N-acetylgalactosaminidase,beta-L-arabinopyranosidase |
| 34442 | 220.5 | GT2 | cellulose synthase,chitin synthase |
| 25682 | 199.3 | GH17 | endo-1,3-beta-glucosidase, licheninase |

Additional table 2. The relative high expression of alpha-amylase genes at the highest temperature stage (N3) and mature stage (N4) of NF liquor starter

| Gene ID | N3-RPKM | N4-RPKM |
| --- | --- | --- |
| 17558  (NFAmy13A) | 293.5 | 30.9 |
| 6731 | 119.1 | 40.4 |
| 15586 | 26.7 | 16.7 |
| 17376 | 26.3 | 4.9 |
| 50650 | 0.0 | 0.4 |
| Total | 465.7 | 93.4 |
